# Establishment of an animal model of immune‐related adverse events induced by immune checkpoint inhibitors

**DOI:** 10.1002/cam4.70011

**Published:** 2024-07-13

**Authors:** Yuan Meng, Yingge Lv, Meng Shen, Wenwen Yu, Yumeng Liu, Ting Liu, Gen Liu, Shiya Ma, Zhenzhen Hui, Xiubao Ren, Liang Liu

**Affiliations:** ^1^ Tianjin Medical University Cancer Institute and Hospital, National Clinical Research Center for Cancer Tianjin China; ^2^ Key Laboratory of Cancer Prevention and Therapy Tianjin China; ^3^ Tianjin's Clinical Research Center for Cancer Tianjin China; ^4^ Key Laboratory of Cancer Immunology and Biotherapy Tianjin China; ^5^ Department of Immunology Tianjin Medical University Cancer Institute and Hospital Tianjin China; ^6^ Department of Biotherapy Tianjin Medical University Cancer Institute and Hospital Tianjin China; ^7^ Haihe Laboratory of Cell Ecosystem Innobation Fund Tianjin China

**Keywords:** animal model, cancer immunotherapy, immune checkpoint inhibitors, immune‐related adverse events

## Abstract

**Objective:**

Immunotherapy, specifically immune checkpoint inhibitors (ICIs), has revolutionized cancer treatment. However, it can also cause immune‐related adverse events (irAEs). This study aimed to develop a clinically practical animal model of irAEs using BALB/c mice.

**Methods:**

Subcutaneous tumors of mouse breast cancer 4T1 cells were generated in inbred BALB/c mice. The mice were treated with programmed death‐1 (PD‐1) and cytotoxic t‐lymphocyte antigen 4 (CTLA‐4) inhibitors once every 3 days for five consecutive administration cycles. Changes in tumor volume and body weight were recorded. Lung computed tomography (CT) scans were conducted. The liver, lungs, heart, and colon tissues of the mice were stained with hematoxylin–eosin (H&E) staining to observe inflammatory infiltration and were scored. Serum samples were collected, and enzyme‐linked immunosorbent assay (ELISA) was used to detect the levels of ferritin, glutamic‐pyruvic transaminase (ALT), tumor necrosis factor‐α (TNF‐α), interferon‐gamma (IFN‐γ), and interleukin‐6 (IL‐6). Mouse liver and lung cell suspensions were prepared, and changes in macrophages, T cells, myeloid‐derived suppressor cells (MDSCs), and regulatory (Treg) cells were detected by flow cytometry.

**Results:**

Mice treated with PD‐1 and CTLA‐4 inhibitors showed significant reductions in tumor volume and body weight. The tissue inflammatory scores in the experimental group were significantly higher than those in the control group. Lung CT scans of mice in the experimental group showed obvious inflammatory spots. Serum levels of ferritin, IL‐6, TNF‐α, IFN‐γ, and ALT were significantly elevated in the experimental group. Flow cytometry analysis revealed a substantial increase in CD3^+^T cells, Treg cells, and macrophages in the liver and lung tissues of mice in the experimental group compared with the control group, and the change trend of MDSCs was opposite.

**Conclusions:**

The irAE‐related animal model was successfully established in BALB/c mice using a combination of PD‐1 and CTLA‐4 inhibitors through multiple administrations with clinical translational value and practical. This model offers valuable insights into irAE mechanisms for further investigation.

## 1 INTRODUCTION

In the realm of immunotherapy, tumor immunotherapy differs from conventional radiotherapy and chemotherapy. It plays an antitumor role by enhancing the efficacy of immune cells in targeting and eliminating tumor cells. Tumor immunotherapy has achieved revolutionary success in clinical practice, among which ICI therapy and adoptive cell therapy (ACT) have been the most studied. Recent studies have shown that immunotherapy results in sustained and effective clinical responses^1^, ^2^, ^3^, ^4^, ^5^ against various carcinomas. At present, PD‐1/PD‐L1 inhibitors plus CTLA‐4 inhibitors are the most widely used ICI therapies, as they can restore T‐cell activity of killing tumor cells. Tumor immunotherapy has been widely used for various malignant tumors and has achieved unprecedented efficacy. At the same time, it has also caused certain adverse reactions known as irAEs. The occurrence of irAE not only affects any organ system of the body, but also may lead to the interruption of treatment, and may also have a long‐term impact on the human body.^6^ Therefore, it is important to establish a suitable animal model for the study of the mechanism of irAE.

Several related studies have explored the establishment of irAE models, but these studies have shown variations in both the types and doses of drugs used, as well as the choice of mouse strains. Notably, a study^7^ evaluated irAEs in preclinical cancer models using Foxp3‐GFP‐DTR mice to reduce immune self‐tolerance. Prolonged Treg depletion in these mice induced a rapidly fatal autoimmune disease, similar to the most severe grade 3/4 clinical irAEs. Foxp3‐DTR mice and different tumor models (breast and colorectal cancer) were used to investigate the mechanism, efficacy, and safety of Treg depletion and monoclonal antibodies targeting PD‐1, TIM‐3, and CD137. In other studies, knockout Pdcd1^−/−^mice were used to mimic the irAE model^8^ of ICI‐induced hepatitis, and Pdcd1^−/−^ mice were treated with CTLA‐4 antibody and/or IDO1 inhibitors. The majority of experimental mice used in these aforementioned studies were genetically modified mice, which are expensive, and the established irAE model is not entirely applicable to irAE caused by ICI. Consequently, the mechanisms identified in such mouse models do not fully explain the clinical irAE phenomenon.

In our research, we employed common inbred BALB/c mice, used the mouse breast cancer (4T1) model, and administered a combination of PD‐1 and CTLA‐4 inhibitors over multiple sessions. It is generally accepted that combi ICI is associated with higher incidence and severity of irAEs than monotherapies.^9^, ^10^ Notably, these two antibodies are commonly used as ICIs in clinical practice. Our aim was to develop a more convenient mouse model of ICI‐related irAEs that closely mirrors clinical manifestations, thereby providing a powerful tool for exploring the phenomenon and mechanism of ICI‐induced irAEs. Simultaneously, our research contributes to a deeper understanding of the occurrence and development of clinical irAEs.

## 2 MATERIALS AND METHODS

### 2.1 Mice

Inbred BALB/c mice, aged 6–8 weeks and female, were obtained from Beijing Sipeifu Company. All mice in this study were housed and maintained at the Laboratory Animal Institute of Tianjin Medical University Cancer Institute and Hospital, following approval by the Animal Ethics Committee of Tianjin Medical University Cancer Institute and Hospital. Clinical symptoms in the mice were assessed, and euthanasia was performed when their clinical symptoms met the predefined ethical criteria.

### 2.2 Cell lines

The BALB/c‐derived breast cancer cell line 4T1 was maintained in RPMI 1640 medium containing 10% FBS and penicillin/streptomycin. The cells were purchased from ACTT.

### 2.3 Experimental mouse model

BALB/c mice were subcutaneously injected with 1 × 10^6^ 4T1 cells into the inguinal region. Tumor growth was measured using a Vernier caliper and the drug was administered when the tumor size reached approximately 30 mm^3^. The mice were intraperitoneally injected with anti‐PD‐1 (RMP1‐14) plus anti‐CTLA‐4 (9D9) antibodies at doses of 250 μg/mouse and 200 μg/mouse, respectively, once every 3 days for a total of 5 injections. Anti‐PD‐1 plus anti‐CTLA‐4 were purchased from Bio X Cell.

### 2.4 Antibodies/flow cytometry

Mouse hepatocyte and pneumonocyte suspensions were prepared. The Zombie NIR Fixable Viability Kit was used to distinguish between dead and live cells. For T‐cell surface staining, antibodies FITC anti‐CD3 (17A2) were used. For macrophage staining, Fc blocking was performed with anti‐CD16/32 antibody on ice, and cell surface staining was performed with antibodies PE/Cyanine7 anti‐F4/80 (BM8), APC anti‐CD11b (M1/70), and PE anti‐CD86 (A17199A). Staining with FITC anti‐CD206 (C068C2) was performed after Fixation Buffer. For MDSC staining, APC anti‐CD11b (M1/70) and FITC anti‐Gr‐1 (RB6‐8C5) were used. For Treg cell staining, APC anti‐CD4 (GK1.5), FITC anti‐CD25 (PC61), and PE anti‐FOXP3 (MF‐14) were used; all purchased from BioLegend. Anti‐FOXP3 staining was performed using the Foxp3/transcription factor staining buffer (Thermo Fisher Scientific). All data were analyzed using the FlowJo V10 software.

### 2.5 Detection of serum

Blood samples were obtained from the mouse orbits and centrifuged at 3000 rpm for 20 min to isolate the serum. Assays were performed according to the manufacturer's instructions. All ELISA kits were purchased from Jianglai Bio.

### 2.6 Histology

Mouse tissues were fixed overnight in 4% paraformaldehyde, routinely processed, embedded in wax blocks, sectioned, and stained with H&E. For tissue scoring, lung, and liver sections were referenced against standards by Mayer et al.^11^ colon sections were referenced against standards by Mayer et al.^12^ and the heart sections were referenced against standards by Joukar et al.^13^

### 2.7 CT imaging

For micro‐CT imaging of mouse lung tissue, mice were anesthetized with 2% isoflurane gas and fixed in the Minerve animal chamber with tape. Isoflurane and air gas mixture was used to maintain anesthesia. Vital signs were monitored using respiratory sensors. Low‐dose CT scanning and image acquisition of the mouse lungs were then performed. Mouse lung CT results were analyzed using OsiriX MD 12.0 software.

### 2.8 Statistical analysis

GraphPad Prism software was used for statistical analysis. The differences between groups were assessed using *t*‐tests. *p* < 0.05 was considered statistically significant, *p* < 0.05 was indicated by (*), *p* < 0.01 was indicated by (**), *p* < 0.001 was indicated by (***), and no statistical significance was indicated by ns.

## 3 RESULTS

### 3.1 PD‐1 inhibitor plus CTLA‐4 inhibitor combination administration induced irAEs in the 4T1 mouse breast cancer model

**FIGURE 1:**
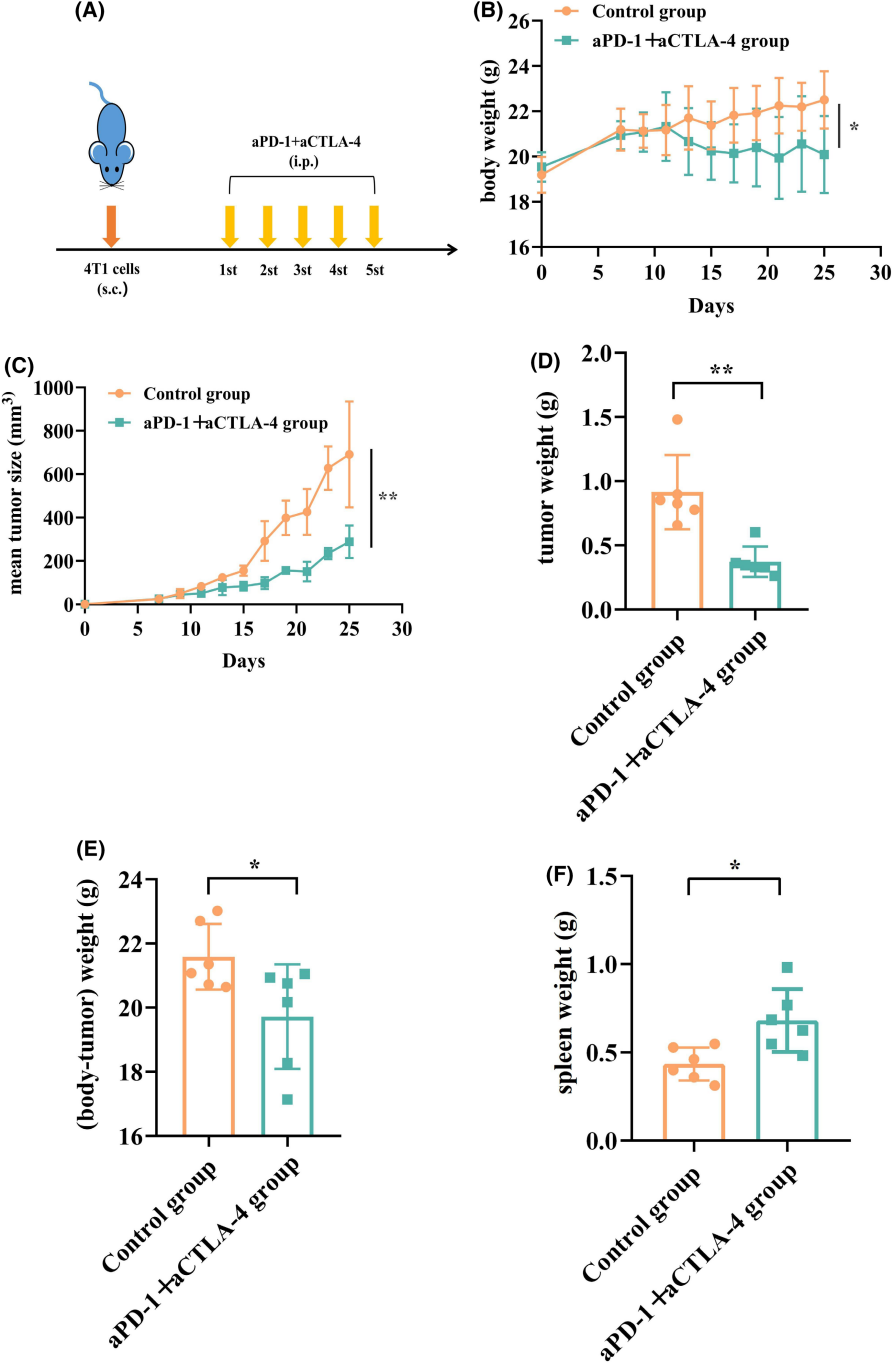
Construction of subcutaneously transplanted 4 T1 tumors in mice and establishment of an immune‐related adverse event (irAE) model. (A) BALB/c mice (*n* = 6/group) were subcutaneously injected with 4T1 tumor cells, and when the tumor reached a mean size of 30 mm^3^ (indicated by arrows), the tumor‐bearing mice were treated intraperitoneally with anti‐PD‐1 plus anti‐CTLA‐4 treatments or PBS once every 3 days for a total of five treatments. (B–D) The body weight, tumor growth, and tumor weight between the control group and aPD‐1 + aCTLA‐4 group. (E) The significant differences between body weight and tumor weight of mice. (F) The significant differences in spleen weight between the group of control and the group of aPD‐1 + aCTLA‐4. *p < 0.05; **p < 0.01.

To investigate ICI‐induced irAEs, we established an animal model of irAE. After in vitro culture of 4T1 mouse breast cancer cells, 6–8 weeks‐old female BALB/c mice were selected to establish a subcutaneously transplanted tumor model with 1 × 10^6^ cells per mouse. When the tumor reached a mean size of 30 mm^3^, aPD‐1250 (μg/mouse) plus aCTLA‐4 (200 μg/mouse) were administered in the experimental group, while the same volume of PBS was given in the control group. The mice received treatment once every 3 days for a total of five administrations (Figure 1A). The body weight of the treatment group was significantly lower than that of the control group about three doses later (Figure 1B). Simultaneously, we found that the tumor volume in the treatment group was significantly lower than that in the control group (Figure 1C), indicating a better therapeutic effect. In order to eliminate the effect of tumor size on weight, we compared the weight of each mouse minus the weight of tumor (Figure 1D), and found that the difference between the weight of the mouse and the weight of the tumor was still lower in the treatment group than in the control group (Figure 1E). Meanwhile, the spleen weight of mice in the treatment group was greater than that in the control group(Figure 1F). In conclusion, the dosage and mode of administration may have caused adverse reactions in mice.

### 3.2 IrAE performances in mouse model

**FIGURE 2:**
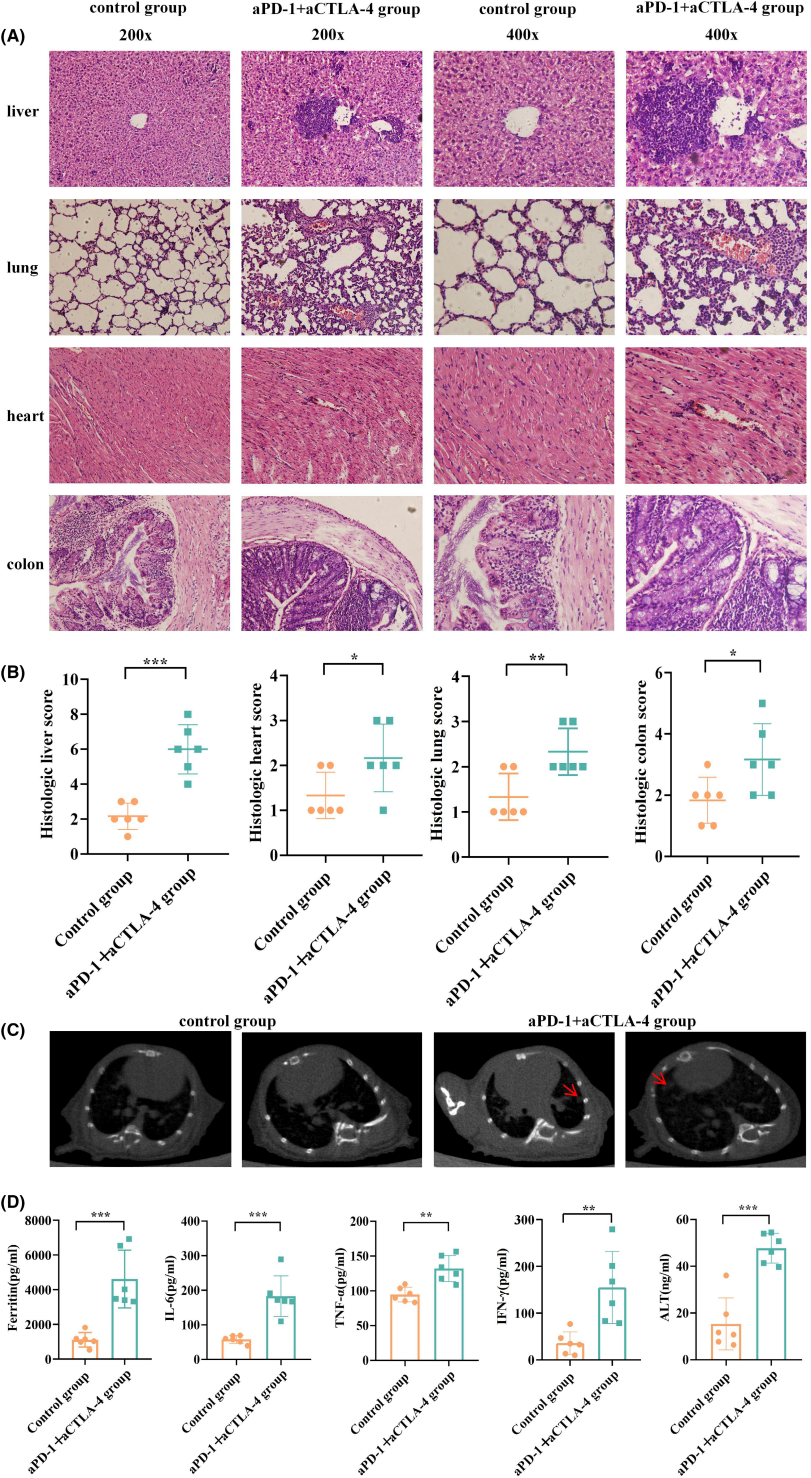
Induction of immune‐related adverse event (irAE) by combination administration in 4T1‐bearing mice. Chest computed tomography (CT) was performed on the day before the last treatment, and liver, lung, heart, and colon tissues, and serum were collected. (A) Representative H&E staining sections of four tissues in the control and aPD‐1 + aCTLA‐4 groups, at 200× (left) and 400× (right). (B) Histological scores of H&E stained sections in two groups. (C) The images of lung CT for mice, the two images on the left were control group, and the two images on the right were aPD‐1 + aCTLA‐4 group. (D) Serum ferritin, IL‐6, IFN‐γ, TNF‐α, and ALT levels in the two groups. Each symbol represents one mouse. *p < 0.05; **p < 0.01; ***p < 0.001.

IrAEs can manifest as systemic responses. To further verify whether the mice had irAEs, the mice were sacrificed, and the liver, lung, heart, and colon of the mice were stained with H&E. Inflammatory infiltration in each organ and tissue of the mice was observed under 200× and 400× magnification, respectively. As shown in the figures, compared with the control group, the experimental group displayed increased inflammatory lymphocyte infiltration in the liver, lung, heart, and colon (Figure 2A). H&E staining of the liver in the experimental group showed obvious hepatocyte degeneration and necrosis, with the disappearance of hepatocyte nuclei. Notably, there was prominent lymphocyte infiltration in the necrotic foci of hepatocytes, along with noticeable lymphocyte infiltration around the central vein of hepatic lobules. In contrast, H&E staining of the control group showed minimal or no lymphocyte infiltration, well‐arranged hepatocytes, and regular liver lobule structures. H&E staining of the lungs in the experimental group showed irregular, shrunken nuclei, damaged connections between adjacent cells, vacuolar cavities, alveolar edema, and enlarged air spaces. In contrast, H&E staining of the control group showed round, centered nuclei, tight intercellular connections, and relatively intact alveolar structures. H&E staining of the hearts showed significantly higher inflammatory lymphocyte infiltration in the experimental group compared with the control group. H&E staining of the colon in the experimental group showed obvious colon damage with substantial inflammatory lymphocyte infiltration and intestinal submucosa edema. In contrast, H&E staining of the colon in the control group showed only mild intestinal mucosal injury and relatively normal epithelial morphology. There were significant differences in the inflammatory scores between the experimental and control groups for all four tissues. The inflammatory scores for the liver were *p* < 0.001, and for the lung, heart and colon, they were *p* < 0.05, indicating varying degrees of hepatitis, pneumonitis, myocarditis, and enteritis in the mice of the adverse reaction model (Figure 2B). This suggests that the model was successfully constructed.

As evident from the H&E staining, lung samples showed significant differences between the experimental and control groups, with obvious pneumonitis in the experimental group. To further confirm our adverse reaction model, we performed lung CT examinations on both groups. The control group showed clear lung fields without abnormal density. In contrast, the experimental group had obvious inflammatory patchy shadows. All mice in the experimental group exhibited varying degrees of abnormal changes on lung CT, consistent with the lung H&E staining results. This indicates that the mice in our constructed adverse reaction model developed pulmonary inflammation (Figure 2C).

The literature shows that cytokines are critical for irAE development, including cytokine storm as an irAE.^14^ Adverse effects associated with ICIs, including irAEs, involve various immune cells, such as T cells, B cells, natural killer cells, macrophages, and endothelial cells. These cells contribute to systemic inflammation via the release of various cytokines. Several studies have shown that IL‐6 plays a central role in this process.^15^ Therefore, we examined the expression of IL‐6, IFN‐γ, and TNF‐α in mouse serum. The results showed that the serum concentration of IL‐6, IFN‐γ, and TNF‐α in the experimental group was significantly higher than that in the control group (Figure 2D). Ferritin in mammals is mainly an intracellular cytosolic iron storage and detoxification protein, whereas in serum, it is an extracellular ferritin that has been widely used in diagnostic tests. In clinical settings, serum ferritin assessment is most commonly used to estimate body iron stores because low serum ferritin is associated with iron depletion, whereas high serum ferritin is associated^16^ with elevated body iron stores or inflammation in patients with normal body iron stores. Serum ferritin is a marker of inflammation, infection, and malignancy. Our previous study showed that ferritin can be used as a diagnostic, differential diagnostic, and prognostic marker^17^ for irAE in patients undergoing ICI treatment. Our current model's results reinforced this finding, revealing significantly higher serum ferritin levels in irAE mice compared with the control group (Figure 2D). Furthermore, pro‐inflammatory cytokines (including IL‐6 and TNF‐α) can directly stimulate ferritin transcription and translation.^18^ To further verify the occurrence of hepatitis in this mouse model, we detected serum ALT related to hepatocyte function. The results showed that serum ALT concentration was significantly higher in the experimental group compared with the control group (Figure 2D), consistent with the liver H&E staining results. This indicates that the mice in our constructed adverse reaction model developed hepatitis. In summary, irAE occurred in various organs in the mouse model we constructed, indicating that the irAE model was successfully constructed.

### 3.3 Changes in immune cell populations in the irAE model

**FIGURE 3:**
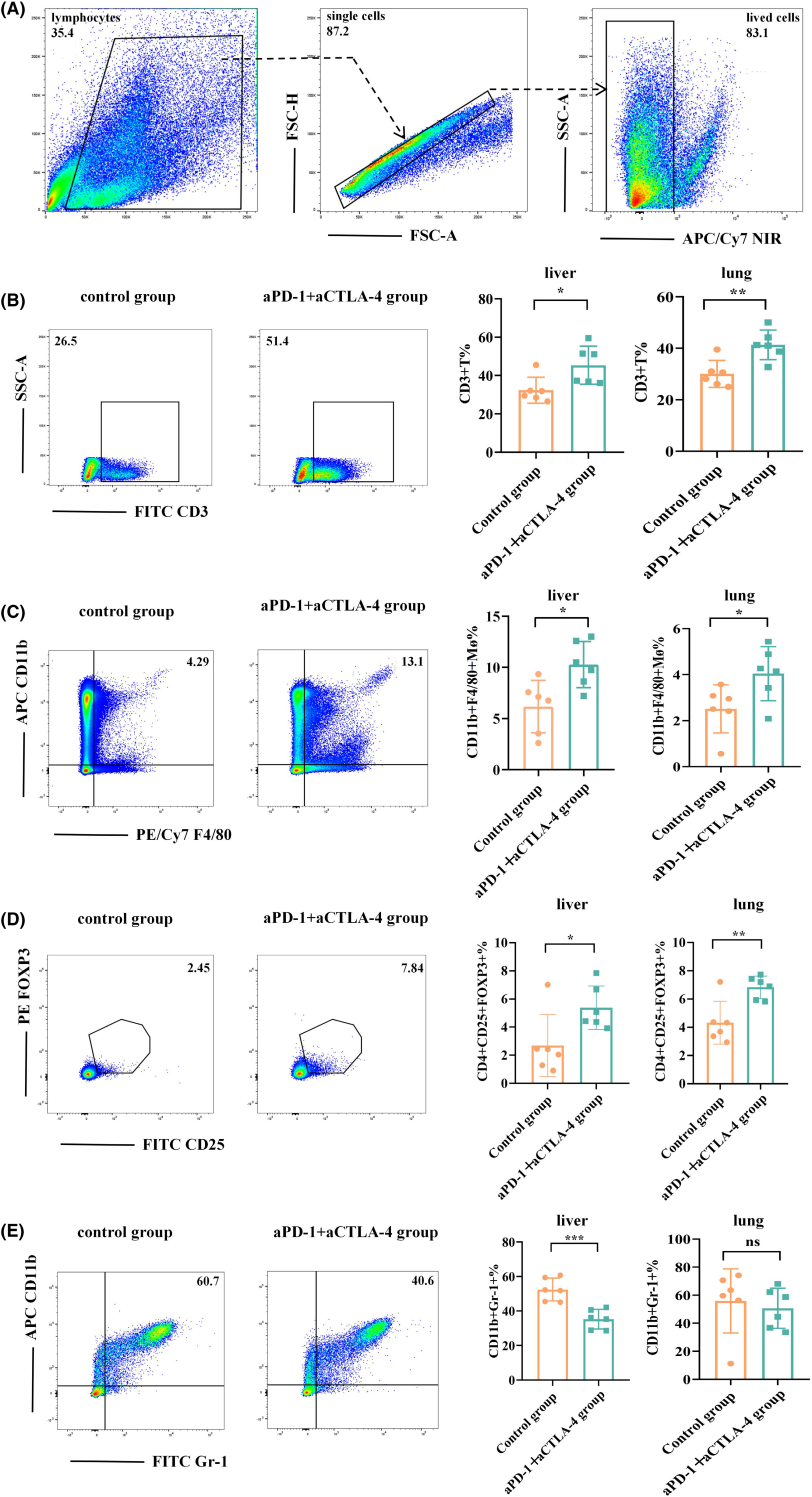
Changes in immune cell populations in tissues in which immune‐related adverse event (irAE) occurred. Single‐cell suspensions of mouse liver and lung tissue were prepared for flow cytometry analysis. (A) Gating strategy for flow cytometry analysis. (B–E) The proportion of CD3^+^ T cells, F4/80^+^CD11b^+^ macrophages, CD4^+^CD25^+^FOXP3^+^ Treg cells, and CD11b^+^Gr‐1^+^ MDSCs. Each symbol represents one mouse. *p < 0.05; **p < 0.01; ***p < 0.001; ns, not statistically significant.

An inflammatory response can manifest at various tissue sites; however, we focused our investigation on the liver and lungs due to their clinical significance as irAE sites. Some studies have shown that T cells play an important role in irAEs.^8^ Therefore, we examined the proportion of T cells in the liver and lungs of mice. Our results revealed a significantly higher proportion of T cells in the experimental group with adverse reactions compared with the control group (Figure 3B). This suggests an important role for T cells in our model, potentially contributing to adverse reactions. This aligns with the conclusions of previous animal model studies on irAEs.^7^, ^8^ Some studies have shown that macrophages play an important role in irAEs.^19^, ^20^ Furthermore, our previous experimental results showed that ferritin also plays an important role in irAEs.^17^ Macrophages and ferritin levels are closely related, as macrophages may be the main source of serum ferritin. Serum ferritin can more specifically reflect the iron status of macrophages, which can also explain the fact that serum ferritin increases during inflammation.^21^ Therefore, we determined the proportion of macrophages in the liver and lungs of mice and found that the proportion of macrophages in the experimental group with adverse reactions was significantly higher than that in the control group (Figure 3C). This underscores the significant role of macrophages in our model, suggesting their potential contribution to adverse reactions.

Tregs suppress immune function through multiple mechanisms, such as CTLA‐4‐mediated inhibition of APC function, IL‐2 consumption, immunosuppressive cytokine production, and immunosuppressive metabolite production.^22^ CTLA‐4 signaling can maintain or control Treg function, allowing them to act as “suppressor cells.” Studies have shown that CTLA‐4 deficiency affects central and peripheral tolerance and Treg cell‐mediated suppression.^23^ In our irAE animal model, a combination of PD‐1 and CTLA‐4 inhibitors was employed. Compared with the control group, Treg cells in the irAE tissues of the treated group did not decrease because of the CTLA‐4 blockade. Instead, the proportion of Treg cells was significantly higher in the treated group than that in the control group (Figure 3D). This may be because the occurrence of irAEs triggerred an increased responsiveness of Treg cells to play a suppressive role and reduce irAE occurrence. The abundance of Treg cells has been demonstrated to increase over time following ICI treatment, and the functionality of peripheral blood Treg cells in systemic inflammation is typically preserved post‐ICI treatment. However, impaired Treg function may contribute to more severe irAE.^24^ Studies have also shown that Treg populations are preserved or even expanded in adverse reactions induced by CTLA‐4 blockade, even when biopsies are performed shortly after symptom onset, suggesting that Treg depletion is not a driving factor.^25^

Immunosuppression is a major feature of MDSCs, which accumulate in chronic inflammatory diseases. In the acute phase of the inflammatory response, TNF‐α can induce the expression of IL‐6, which in turn leads to the upregulation of several acute‐phase response factors, and IL‐6 also plays a role in MDSC accumulation.^26^, ^27^ MDSCs can directly inhibit the antitumor functions of T and NK cells by expressing reactive substances and immune checkpoint.^28^ Our results showed that the number of MDSCs in irAE mice was lower than that in control mice (Figure 3E). This result may be due to the blocking of PD‐1 and CTLA‐4 in mice in the experimental group, which further reduces MDSC.

## 4 DISCUSSION

Several studies have been conducted to explore the construction of irAE animal models (Table 1). In a 2021 study, Llewellyn et al.^8^ developed a novel mouse model. In Pdcd1 mice, anti‐CTLA‐4, and/or IDO1 inhibitors or 4‐1BB agonist antibodies were administered. In a study of immune‐mediated hepatotoxicity related to various immunotherapies, Marschner et al.^29^ found that miR‐146a played an important role in irAE occurrence. They established an irAE animal model by combining anti‐PD‐1 plus LPS treatment in miR‐146 knockout mice. The inflammatory target organs in this animal model included the lungs, liver, and colon. Another study^30^ used DSS to pre‐induce colitis, then used anti‐PD‐1 plus anti‐CTLA‐4 treatment to aggravate colitis to construct an adverse reaction model. In 2016, Liu et al.^7^ also used various inhibitors to study the irAEs in various target organs. In addition to the abovementioned irAE model studies, there is also a monkey model study^31^ characterized by multiple organ toxicities, including myocarditis. Chinese cynomolgus monkeys were intravenously injected with a vector or nivolumab plus ipilimumab to establish the model. Notably, only a few studies have been conducted on irAE model construction, and most of the published research has relied on genetically modified mice. These genetic mouse models may involve extended production cycles and high associated costs. Furthermore, the drugs used to construct these models are not simple ICI treatments. In contrast, our model used common inbred BALB/c mice and clinically relevant ICI drugs, making it a convenient choice for research. This selection provides us with the advantages of convenience and clinical relevance. The current study, however, has a limitation in that it lacks a comparative analysis of mice with varying genetic backgrounds and ages. Bauché et al.^32^ demonstrated that long‐term treatment with an Fc‐effector anti‐CTLA4 antibody twice weekly in Balb/c mice induces enterocolitis, and C57BL/6 mice were less prone to developing Fc‐effector anti‐CTLA4‐mediated colitis compared with Balb/c mice, and monotherapy anti‐PD‐1 treatment didn't elicit gut inflammation. In the study of Tsukamoto et al.^33^ the serologic parameters, multiple organs including lung, kidney, and liver from ICB‐treated aged mice, but not from their younger counterparts that showed irAEs. The aforementioned studies have provided theoretical support for the impact of age and different genetic background on irAE. Model construction such as C57BL/6 mice, which are also inbred strains, was not performed, due to the consideration that C57BL/6 mice tend to be more active and are mostly used for creating genetically modified mice. C57BL/6 mice are widely used in tumors, biology, genetics, and other fields and are commonly used in various animal models, such as type 2 diabetes mellitus.^34^ In contrast, BALB/c mice are known for their docility, high tumor susceptibility, and extensive use in tumor and immunology studies, making them suitable for modeling viral or bacterial infections.^35^ Therefore, we first constructed a BALB/c mouse model, and the experimental results confirmed the success of the model. In future studies, we will consider adding a C57BL/6 mouse model.

**TABLE 1:** Research progress on immune‐related adverse events (irAEs) animal model construction.

Serial number	Medicines	Species	Inflamed organs	Reference
1	Anti‐CD137 antibody; Anti‐PD‐1 antibody; Anti‐CTLA4 antibody; Anti‐TIM‐3 antibody	C57BL/6 Foxp3‐DTR‐GFP mice; BALB/c Foxp3‐DTR‐GFP mice	Liver; Colon; Eye lid	Liu et al.^7^
2	Anti‐CTLA‐4 antibody; IDO1 inhibitor; Anti‐(4‐1BB) agonistic antibody	Pdcd1 mice	Liver	Llewellyn et al.^8^
3	Anti–PD‐1 antibody; LPS	miR‐146a^−/−^ mice (C57BL/6 background)	Lung; Liver; Colon; Skin	Marschner et al.^29^
4	Anti–PD‐1 antibody; Anti‐CTLA4 antibody; DSS	Rag2^−/−^Il2rg^−/−^, OT‐I and Pmel‐1 mice; C57BL6 mice	Colon	Perez‐Ruiz et al.^30^
5	Nivolumab; Ipilimumab	Cynomolgus Monkeys	Heart	Ji et al.^31^
6	Combining Fc‐null or Fc‐mutant CTLA4 antagonists with PD‐1 blockade	C57BL/6J and Foxp3‐GDL mice; Wild‐type Balb/c mice; C.129S6(B6)‐Rag2tm1Fwa N12 mice; C.B‐17 scid mice	Small intestine; Colon	Bauché et al.^32^
7	Anti–PD‐1/PD‐L1 Abs	C57BL/6J(IL‐6–deficient, IL‐21–deficient, and IFN‐γ–deficient) mice	Lung; Liver; Kidney	Tsukamoto et al.^33^

The mouse model constructed in this study exhibits a significant correlation with the clinical performance of irAEs. Firstly, considering the site of irAE manifestation, studies have demonstrated^36^ that different immune checkpoint inhibitors exhibit distinct toxicity profiles; for instance, CTLA‐4 inhibitors commonly result in colitis, encephalitis, and rash at various levels. Conversely, PD‐1 inhibitors are more frequently associated with pneumonitis, hypothyroidism, joint pain, and vitiligo. Our model aligns with these findings as we observed colitis and pneumonitis through HE staining and lung CT scans respectively. Secondly, by examining inflammatory cytokines and other related markers in peripheral blood samples; research has indicated a close relationship between IL‐6/TNF‐α levels and irAE.^37^ Antagonists targeting these cytokines have also been employed clinically for treating irAEs. In our model experimental group, mice exhibited significantly higher serum levels of both cytokines compared with other mice groups. In histological manifestations, the study^38^ focuses on immune checkpoint inhibitor‐induced hepatotoxicity in seven patients, revealing elevated liver enzyme levels in all patients. Histologically, all biopsies showed predominantly lobular hepatitis with milder portal inflammation. Immunostaining revealed the presence of large numbers of CD3^+^ and CD8^+^ lymphocytes. In our study, the presence of ALT was also observed, indicating a significant increase in ALT levels among mice in the administration group. Additionally, histological examination using HE staining revealed extensive lymphocyte infiltration and inflammation in the portal vein. The incidence of immune checkpoint inhibitor pneumonitis was observed in 44 out of 299 patients, as reported by another study.^39^ All patients who developed chronic ICI pneumonitis had bronchoalveolar lavage fluid (BALF) lymphocytosis on cell differential and organizing pneumonia on lung biopsy at initial ICI pneumonitis presentation. The imaging findings serve as clinical evidence confirming the presence of pneumonitis. Our study further demonstrated that mice in the administration group exhibited signs of lung inflammation on CT scans, while histological examination using HE staining revealed increased lymphocyte infiltration and other related changes. Additionally, our previously published results^17^ on ferritin as an irAE marker were further validated using this mouse model study.

It has been shown that T cells and macrophages also play important roles in irAEs. Llewellyn et al.^8^ used combined blockade of PD‐1, CTLA‐4, and IDO1 to establish a mouse model of ICI‐induced hepatitis and studied the immune mechanism in this model. The infiltration of liver tissue was found to be dominated by CD4^+^ and CD8^+^ T cells, and T‐cell depletion abolished ICI‐mediated hepatitis. Single‐cell sequencing revealed that IFN‐γ and hepatic monocyte‐derived macrophages play central roles in promoting pro‐inflammatory T‐cell responses to ICI combinations. In our model, experimental group mice exhibited significantly higher serum levels of IFN‐γ compared with other mice groups. Our flow cytometry results also showed that the proportions of T cells and macrophages in the liver and lungs of the PD‐1 inhibitor plus CTLA‐4 inhibitor group were significantly higher than those in the control group, further confirming the important role of T cells and macrophages in the organs and tissues where adverse reactions occur. However, the cell populations, whether T cells and macrophages are consistently involved, the factors and pathways enabling cell interactions, and the further establishment of organ‐specific irAE models to explore their mechanisms need to be further confirmed by in vivo and in vitro experiments.

In recent years, classical (M1 type) and alternative (M2 type) activation of macrophages has been increasingly recognized. M1 macrophages are activated by the “pro‐inflammatory” cytokine spectrum, namely IL‐6 and TNF‐α. M2 macrophages are associated with some processes related to immune downregulation but are less related to the secretion of cytokines related to the development of inflammation.^40^ Therefore, the function of macrophages in the immune system may be related to their roles in immunosuppression and immune regulation. Macrophages are associated with ICI‐related irAE,^19^, ^20^ and serum ferritin levels, and some studies have shown that macrophages may be the main cellular source of serum ferritin, and that serum ferritin can more specifically reflect the iron status of macrophages.^21^ This may also explain why serum ferritin levels are elevated during inflammation. Serum ferritin primarily consists of L‐ferritin, which is released from the liver. In addition, several inflammation‐related cytokines, such as IL‐6 and TNF‐α, have been shown to stimulate ferritin transcription and translation,^18^ which also explains the high ferritin levels seen under inflammatory conditions. In summary, the secretion of serum ferritin, which has been shown to play a predictive role in irAEs, is related to macrophages, which are also upregulated in the organs and tissues where irAEs occur. Whether macrophages exacerbate ICI‐induced irAEs through ferritin secretion remains unclear. These hypotheses need to be further tested and confirmed by in vitro and in vivo experiments.

The establishment of this model not only offers a clinically relevant mouse model for studying ICI‐related irAEs, but also provides a theoretical foundation for further investigating the mechanisms underlying irAEs, exploring treatment strategies that can prevent, mitigate or reverse irAEs without compromising tumor therapeutic efficacy, and facilitating future research on the correlation between different tumor types and irAEs.

## 5 CONCLUSIONS

In conclusion, a mouse model of irAEs was established using a combination of PD‐1 and CTLA‐4 inhibitors. Following treatment, the mice exhibited significant weight loss, increased inflammatory infiltration in organs and tissues, and patchy inflammatory shadows on lung CT. Elevated levels of the inflammatory cytokines, IL‐6, and TNF‐α, as well as the liver enzyme ALT, were also detected in the serum of treated mice compared with controls. Together, these findings demonstrate that administration of PD‐1 and CTLA‐4 inhibitor combinations can successfully induce irAEs in inbred mouse BALB/c, which may help further elucidate the mechanisms underlying these clinically relevant irAEs. Future research should explore diverse mouse strains, investigate immune cell interactions, delve into ferritin's role, and translate findings for clinical applications.

## AUTHOR CONTRIBUTIONS

**Yuan Meng:** Conceptualization (equal); writing – original draft (equal). **Yingge Lv:** Methodology (equal). **Meng Shen:** Data curation (equal). **Wenwen Yu:** Methodology (equal). **Yumeng Liu:** Methodology (equal). **Ting Liu:** Methodology (equal). **Gen Liu:** Methodology (equal). **Shiya Ma:** Methodology (equal). **Zhenzhen Hui:** Data curation (equal). **Xiubao Ren:** Writing – review and editing (equal). **Liang Liu:** Writing – review and editing (equal).

## FUNDING INFORMATION

This study was funded by the National Natural Science Foundation of China (82372779, 82303196, 82373279, 82373283, U20A20375, 82103001, and 82302913), Haihe Laboratory of Cell Ecosystem Innovation Fund (22HHXBSS00004), and Tianjin Key Medical Discipline (Specialty) Construction Project (TJYXZDXK‐009A), and by the Tianjin Natural Science Foundation (21JCQNJC01430).

## CONFLICT OF INTEREST STATEMENT

This study has not been presented in anywhere. The authors reported no potential conflicts of interest.

## ETHICS STATEMENT

All experiments involving mice were approved by the Animal Ethical and Welfare Committee of Tianjin Medical University Cancer Institute and Hospital.

## Data Availability

The data that support the findings of this study are available from the corresponding author upon reasonable request.
